# Ethnicity and cancer in Guyana, South America

**DOI:** 10.1186/1750-9378-4-S1-S7

**Published:** 2009-02-10

**Authors:** Wallis S Best Plummer, Premini Persaud, Penelope J Layne

**Affiliations:** 1 The Cancer Registry of Guyana, Georgetown, Guyana

## Abstract

**Background:**

The Cancer Registry of Guyana, a population-based registry was established in 2000. Over the past eight years, data has been collected from the national referral hospital and other public and private institutions.

**Methods:**

A comprehensive review of the Registry's database was undertaken, focusing on the ethnic and site prevalence of the three major reported cancers. The data was then subjected to summary statistics and the frequencies of cases by ethnicity and age-group were subjected to chi squared analysis. A 0.05 level of significance was applied to all tests.

**Results:**

There was a clear ethnic distribution of the three major cancers (breast, cervical and prostate) within the database. Afro-Guyanese men accounted for over 65% of prostate cancers. Among women, Indo-Guyanese presented with the most cases of breast cancer (45%) while Afro-Guyanese had the majority of cervical cancer cases (39%). When the proportion of cervical cancer cases for all cancers in an ethnic group was analysed however, cervical cancer was significantly more common (p < 0.0001) among Indigenous Amerindian women. Similarly, by age-group analysis, there were significantly more cases of cervical than breast cancer (p = 0.014) among women under 30 years of age.

**Conclusion:**

The Cancer Registry of Guyana reflects a high incidence of prostate, cervical and breast cancers among Afro-Guyanese. Socio-economic, dietary and genetic influences on the observed pattern of incidence within this ethnic sub-group, as well as those of Indo-Guyanese and Indigenous Amerindians warrant further investigation.

## Background

Guyana is the only English-speaking country within South America, lying on the northern Atlantic coast between Venezuela and Surinam. The ethnic distribution of the population reflects the influences of the periods of slavery and indentureship as 44% are of East Indian and 30% of African ancestry. A further 9% are Indigenous Amerindian with the remainder of the population being of mixed race, European or Chinese. According to the last census report [1], the population of 751,223 persons was almost evenly distributed between the genders (50.1% male), with a median age of 23 years.

Cancer has been one of the five leading causes of death reported by the Statistical Unit of the Ministry of Health in Guyana over the last fifteen years. The population-based Cancer Registry of Guyana, which is an active part of the Ministry of Health's national disease surveillance system, was established in 2000, and has since been providing policy makers with reliable cancer data based upon pathologically-supported diagnosis acquired through a system of data abstraction from the records of public and private health care institutions and laboratories across the country. Ethnic trends in cancer diagnoses in Guyana have been alluded to for some time [2,3]. In this context, studies in other countries have consistently reported the disproportionate prevalence of prostate cancer in men of African descent [4] and the severity/mortality of breast cancer in women of African descent [5,6]. Similarly, studies with Indian/South Asian men and women have also described the higher prevalence of breast and prostate cancers in migrant (US based) populations [7] compared with native Indians who have higher rates of oral cancer (males) and cervical cancer (females), Further, a high prevalence of cervical cancer has been reported among Indigenous people of both Surinam [8] and Queensland Australia [9]. Considering the ethnic basis of the Guyanese population, the Registry's data was reviewed to assess the relevance of these trends.

## Methods

The Registry's database was sorted by ethnicity and site prevalence, and then summary statistical analyses were done to highlight the frequencies of cases by gender and age. The significance of the differences in the incidence of the three main cancers (breast, cervical, and prostate) according to ethnicities and age-group was also determined by chi squared analysis followed by Fisher exact test at the 0.05 level of significance.

## Results

The database represented all diagnoses and deaths due to cancer collected from all public health facilities across Guyana and private facilities. It also included those Guyanese who had sought cancer treatment in Trinidad between 2000 and 2006. Within the database, the three main cancers were breast (23%), cervical (22%) and prostate (20%). Afro-Guyanese males accounted for over 65% of prostate cancer cases. Afro-Guyanese women presented with the highest number of cervical cancer cases while the number of Indo-Guyanese women with breast cancer was the highest (Table 1). The significance of the differences in the relative numbers of each cancer by ethnic group as represented by the population in the Registry's database was determined. There was no significant difference between cervical and breast cancer incidence of Afro- and Indo-Guyanese women in the Registry. However, cases of cervical cancer were significantly higher among Indigenous Amerindians (p < 0.0001) than among either the Afro- or Indo-Guyanese Registry populations. Similarly, cases of prostate cancer among Afro-Guyanese males were significantly higher (p < 0.0001) than those of Indo- or Amerindian males within the Registry.

**Table 1:** The distribution of the three main cancers within the Cancer Registry of Guyana by ethnicity.

Ethnicity (N)	Breast	Cervical	Prostate
Afro-Guyanese (1215)	256	225	341**
Indo-Guyanese (1161)	265	213	101
Amerindian (117)	8	57*	12
Other/not stated (276)	60	78	71
TOTAL	589	573	525

The table shows the comparative prevalence of the three principal cancers within the main ethnic subgroups of the Guyanese population. * p < 0.0001, ** p < 0.0001.

**Figure 1:**
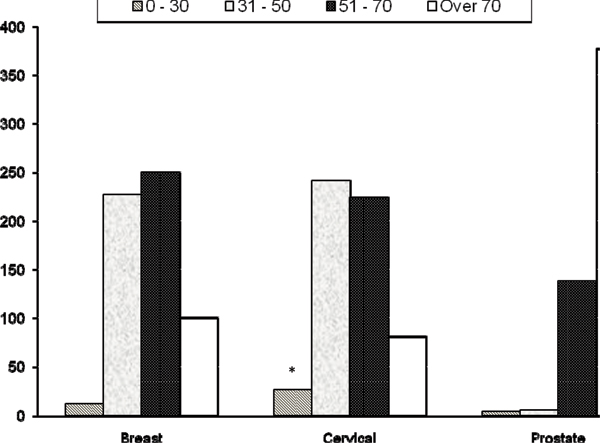
**The distribution of three main cancers by age-group within the Cancer Registry of Guyana**. *Incidence cervical cancer significantly different from breast cancer in 0–30 age group (p = 0.014).

Age group analysis showed a predominance of prostate cancer (49.9%) in the male population over 70 years of age (Figure 1). Likewise, there were significantly more cases of cervical than breast cancer among women under 30 years of age (p = 0.014). In comparison, while the majority of cervical and breast cancer cases were found in women between 31 and 70 years of age (81%), there was no significant difference in the incidence of breast or cervical cancer between the age-groups 31–50, 50–70 and over 70 years. In terms of the timeliness of diagnosis, breast and cervical cancers were generally diagnosed earlier (stages 1 and 2) than those of other sites. Unfortunately, the majority of prostate cancers were not staged.

## Discussion

There are several challenges to Registry work in Guyana. These include the absence of a Cancer Act making cancer a notifiable disease, the failure to highlight cancer as a contributory cause of death where it is the underlying disease and diagnosis/treatment-seeking by affluent Guyanese outside of Guyana. Although the Registry captures all cases diagnosed and treated in Guyana, undiagnosed cases and deaths due to cancer that were not managed by a health care worker (estimated at less than 10%), are inevitably lost to the Registry.

The high incidence of the various cancers among Afro-Guyanese was not without precedence, as a pilot study of cancer care in Guyana in the early 1990s had previously found a 54% prevalence of cancer among this ethnic sub-population of the sample studied [2]. Likewise, a high incidence of cervical cancer among Indigenous Amerindian women has been recognised previously [3], although, in comparison, the high number of cervical cancer cases among Afro-Guyanese women was unexpected. Since the Afro-Guyanese and Indo-Guyanese constitute 74% of the national population however, it was not surprising that over 85% of all cases were found among persons in these ethnic groups.

There are several likely determinants of the ethnicity-based incidence of prostate, breast and cervical cancer in the Registry. Both the high incidence of prostate cancer among men of African descent [4], and the higher incidence of prostate cancer among US based-compared with native Indians has also been recognised [7]. As reported from neighbouring Trinidad and Tobago for their residents, [4] a three-fold higher incidence of prostate cancer among the Guyanese of African-compared with Indian-descent was seen. Apart from genetic determinants, these findings allude to a role for dietary, cultural and environmental influences in the observed trends of prostate cancer.

Several demographic characteristics associated with a higher risk for cervical cancer have been highlighted among Indigenous Amerindians in Guyana. These include a mean of 4.5 babies per woman (range 0–14; n = 672) and a mean age at first intercourse of 16.8 years (range 9–26; n = 559). Additionally, among 412 women screened, there was a 22.8% prevalence of high risk HPV [3]. These factors along with the low socio-economic status of this population and limited access to health care can explain aspects of the high incidence of cervical cancer in this ethnic sub-group of the Registry. Unfortunately although comparable data is not yet available on the Afro-Guyanese sub-population, factors associated with this group such as low income and socioeconomic status, being overweight, and diet are likely to be key determinants of the observed incidence.

An earlier study of the prevalence of risk factors including age at menarche, age at menopause, age at first pregnancy, lactation, use of oral contraceptives and hormone replacement therapy, first-degree relatives with breast cancer, history of benign breast lumps/cysts, smoking, alcohol use and exercise, among a group of Guyanese breast cancer patients (60% Indian descent and 30% African descent), failed to identify any risk factor or cluster of factors associated with the prevalence of breast cancer in either ethnic sub-group[10]. Rather, the sample was characterised by a predominance of risk-reducing factors, reaffirming the previously reported observation of an absence of identifiable breast cancer risk factors among many patients [11]. In this context, the impact of genetic susceptibility and still unidentified environmental exposures is likely to be important.

## Conclusion

Clearly ethnic trends underlie the prevalence of the three main cancers among Guyanese in the database of the Cancer Registry of Guyana. While various socioeconomic, dietary, obesity-related, genetic and environmental factors are likely to be driving the ethnic distribution of the cancers, these will need to be studied further to assess their relative impacts.

## Competing interests

The authors declare that they have no competing interests.

## Authors' contributions

This manuscript was prepared by Dr. Wallis Best Plummer based upon a concept developed collaboratively with Mrs. Penelope Layne; invaluable support for data analysis was provided by Ms. Premini Persaud.
